# CTRP3 alleviates cardiac ischemia/reperfusion injury via LAMP1/JIP2/JNK signaling pathway

**DOI:** 10.18632/aging.203876

**Published:** 2022-02-03

**Authors:** Yanbin Song, Yunqing Zhang, Zhaofei Wan, Junqiang Pan, Feng Gao, Fei Li, Jing Zhou, Junmin Chen

**Affiliations:** 1 Department of Cardiovasology, Yan’an University Affiliated Hospital, Yan’an 716000, China; 2 Department of Pathology, Yan’an University Affiliated Hospital, Yan’an 716000, China; 3 Department of Cardiology, The Second Affiliated Hospital of Xi’an Jiaotong University, Xi’an 710038, China; 4 Department of Cardiology, Xi’an Central Hospital, Xi’an 710061, China

**Keywords:** ischemia/reperfusion injury, oxygen-glucose deprivation/reoxygenation, CTRP3, LAMP1, oxidative stress

## Abstract

Background: C1q/tumor necrosis factor-related protein 3 (CTRP3) has been reported to be a crucial regulator in myocardial infarction. Nevertheless, the potential molecular mechanism of CTRP3 in ischemia/reperfusion (I/R) injury remains largely unclear.

Methods: The cell model of myocardial I/R injury was established by oxygen-glucose deprivation/reoxygenation (OGD/R) of rat cardiomyocyte H9C2. Expression of CTRP3 and lysosomal-associated membrane protein 1 (LAMP1) was detected in H9C2 cells treated with oxygen-glucose deprivation/reoxygenation (OGD/R). H9C2 cells were transfected with overexpression plasmids of CTRP3 (pcDNA-CTRP3) and LAMP1 (pcDNA-LAMP1), or CTRP3 small interfering RNA (si-CTRP3) or/and pcDNA-LAMP1, and cell proliferation, apoptosis and oxidative stress were testified. Co-IP assay was performed to validate the relationship among CTRP3, LAMP1 and JIP2. The role of CTRP3 and LAMP1 in JIP2/JNK pathway was evaluated with Western blot assay. Furthermore, *in vivo* myocardial I/R injury model was constructed to investigate the effect of CTRP3.

Results: Overexpression of CTRP3 and LAMP1 both significantly promoted cell proliferation, inhibited apoptosis and the production of reactive oxygen species (ROS), malondialdehyde (MAD) and cardiac troponin (cTn-I), while silencing CTRP3 exerted the opposite effects, and LAMP1 overexpression reversed the effect of silencing CTRP3 on the aspects above. CTRP3 interacted with LAMP1, and both CTRP3 and LAMP1 bound with JIP2. SP600125 (JNK inhibitor) could restore the effects of CTRP3 or LAMP1 overexpression on the expression of JIP2 and phosphorylated-JNK (p-JNK), proliferation and apoptosis. Moreover, overexpression of CTRP3 improved cardiac I/R injury *in vivo*.

Conclusion: CTRP3 alleviates cardiac I/R injury by elevating LAMP1 and activating JIP2/JNK signaling pathway, which may serve as a potential therapeutic target for I/R injury.

## INTRODUCTION

Myocardial ischemia caused by coronary artery occlusion leads to increased loss of cardiomyocytes and is a main cause of elevated morbidity and mortality in myocardial infarction [1]. Reperfusion of ischemic myocardium is currently the common treatment for patients with acute myocardial infarction. However, the sudden restoration of blood flow may lead to the overproduction of reactive oxygen species (ROS), which promote oxidative stress, resulting in myocardial cell death after ischemia and aggravating myocardial injury [2]. Oxidative stress-induced myocardial injury is not only associated with the increase of ROS, but also related to the decline in the antioxidant defense system. Malondialdehyde (MDA) is a putative marker of lipid peroxidation and a common parameter determining increased free radical formation in post ischemic tissues [3]. Cardiac calponin I (cTn-I) is a contractile protein that is present only in cardiac muscle and serves as a specific biomarker of myocardial injury [4]. At present, the main methods to reduce myocardial I/R injury are myocardial ischemia preconditioning (IP) and post-ischemia conditioning (PIC) [5]. However, IP and PIC are not easy to operate in the clinic. Therefore, it is necessary to explore new strategies for I/R protection of cardiomyocytes to improve the clinical prognosis of patients with ischemic heart disease.

C1q tumor necrosis factor (TNF)-related protein-3 (CTRP3) belongs CTRP family and is a newly found group of proteins homologous to adiponectin (APN) collateral system [6]. It is mainly expressed in adipose tissues and is also found in the heart and liver [7, 8]. CTRP3 has a variety of physiological functions, including reducing blood glucose [9], inflammation [10] and blood lipid [11]. Abnormal expression of CTRP3 is associated with the pathogenesis of many diseases including severe acute pancreatitis [12], diabetic retinopathy [13], and depression [14]. Recently, increasing evidence has indicated that CTRP3 plays an important role in many cardiovascular diseases. Ma et al. found that CTRP3 protected against diabetic cardiomyopathy via activation of the AMPKα pathway [15]. Chen et al. indicated that CTRP3 could efficiently restrain inflammatory response and endothelial dysfunction in atherosclerosis [16]. Yi et al. demonstrated that CTRP3 could promote angiogenesis and inhibit cell apoptosis in ischemic mouse heart [7]. Nevertheless, the involvement of CTRP3 in the pathogenesis of myocardial I/R injury still poorly understood.

Lysosomal associated membrane protein 1 (LAMP1) is a glycosylated lysosomal membrane protein that belongs to lysosomal associated membrane protein family and could protect the lysosomal membranes from intracellular proteolysis [17]. Chen et al. suggested that Ubiquitin-like protein 4A (UBL4A) was low expressed in pancreatic ductal adenocarcinoma, while LAMP1 was positively correlated with UBL4A, and UBL4A exerted antitumor effects on autophagy-related proliferation and metastasis in pancreatic ductal adenocarcinoma (PDAC) by upregulating LAMP1 [18]. Lamp1 positive astrocytes significantly suppress central nervous system inflammation by inducing T cell apoptosis [19]. Tanshinone IIA could improve heart function by upregulating LAMP1 expression and restoring autophagic flux [20]. Interestingly, cilostazol protected against myocardial ischemia by increasing LAMP1 expression [21]. Therefore, LAMP1 might function as a crucial regulator of myocardial I/R injury.

Herein, this study aimed to clarify the effects of CTRP3 and LAMP1 on myocardial I/R injury and the underlying mechanisms, with a view to providing biomarkers for clinical prevention of myocardial I/R.

## METHODS

### Cell culture and OGD/R model establishment

The rat embryonic cardiomyocytes H9C2 were got from the cell bank of Shanghai Biology Institute, Chinese Academy of Science (Shanghai, China) and maintained in Dulbecco’s modified Eagle medium (DMEM, Gibco, Grand Island, NY) supplemented with 10% fetal bovine serum (FBS) in a humidified condition containing 5% CO_2_ at 37°C (Thermo, Waltham, MA, USA). To establish the model of I/R injury *in vitro*, cells were cultured in glucose-free DEME at 37°C in a hypoxic incubator (94% N_2_, 5% CO_2_ and 1% O_2_) for 24 h. After oxygen-glucose deprivation, cells were grown in complete medium at normal incubator (95% O_2_, 5% CO_2_) for 6 h.

### Cell transfection

CTRP3 and LAMP1 overexpression plasmid (pcDNA-CTRP3 and pcDNA-LAMP1), small interfering RNA targeting CTRP3 (si-CTRP3) and LAMP1 (si-LAMP1), as well as their negative control (vector and scramble) were constructed by Genepharma Biotech (Shanghai, China). H9C2 cells were transfected with above plasmid vector with lipofectamine 2000 (Invitrogen, Carlsbad, CA). After transfection for 48 h, cells were given OGD/R treatment, and then cells were collected for subsequent experiments.

### Quantitative real-time polymerase chain reaction (RT-qPCR)

Total RNA from cells was extracted with TRIzol reagent (Invitrogen, Carlsbad, CA, USA) following the manufacturer’s instructions. A TaqMan Reverse Transcription Kit (Qiagen, Valencia, CA) was used to reversely transcribe RNA to complementary DNA. RT-qPCR was run on a 7000 ABI System by using SYBR Green PCR Kit (TaKaRa, Tokyo, Japan). GAPDH was used as an internal control. The relative quantitative expression was determined by using the 2^−ΔΔCT^ method. The primer sequences were described as follow: CTRP3, F, 5′-AGC GTC TCT GGG TTG TCT TG-3′; R, 5′-TGA GCT TCT GCC CCA ACA AA-3′; LAMP1, F, 5′-AGG CGG TGA GAT CTA GAC GA-3′; R, 5′-AAG AAT AGT GTT GGC GGG GG-3′.

### MTT assay

Cell viability was detected by using MTT assay. H9C2 cells were seeded at 5 × 10^3^ cells/well on 96-well plates. After OGD/R treatment, cells were incubated with 20 μL of MTT solution (0.5 mg/mL) for 4 h at 37°C. Then, the supernatant was removed, and 150 μL of dimethyl sulfoxide were added to each well. The absorption was measured at 490 nm by using microplate reader (BioTek Instruments, Inc.).

### Ethynyl deoxyuridine (EdU) incorporation assay

EdU detection was performed by using an EdU immunofluorescence staining kit (Ribobio, Guangzhou, China) according to the manufacturer’s protocol. Sterilized slides were put into 24 well plates, and H9C2 cells were seeded into each well at a density of 3 × 10^4^ cells/well. Transfected cells were incubated with 20 μmol/L EdU reagent for 2 h, washed twice with phosphate Buffer solution (PBS) and fixed with 4% phosphate buffered paraformaldehyde for 15 min. Subsequently, the cells were stained with 100 μL of fresh Apollo reaction cocktail, and nuclei were stained with 100 μL of Hoechst 33342. The percentage of EdU positive cells was counted by using fluorescence microscope (LEICA).

### Flow cytometry analysis

Apoptosis of H9C2 cells was detected by using the flow cytometry and a Annexin phycoerythrin/7-aminoactinomycin D (V-PE/7-AAD) Apoptosis Detection Kit (Vazyme, Nanjing, China). After treatment, cells were collected and digested with trypsin without ethylenediamine tetraacetic acid. Then, the cells were washed three times with cold PBS and resuspended in 100 μL of binding buffer. The cell suspension was incubated with 5 μL of Annexin V-PE and 5 μL of 7-AAD staining solution at room temperature for 10 min in a dark room. The apoptotic ratio of H9C2 cells was assessed within 1 h by using flow cytometry (BD, Franklin Lakes, NJ, USA).

### Western blot analysis

Total protein was extracted with RIPA lysis buffer. Protein concentration was determined by using the Bradford method. Equal amounts of protein were electrophoresed by 15% sodium dodecyl sulfate-polyacrylamide gel electrophoresis and transferred to polyvinyl difluoride (PVDF) membranes. The membranes were blocked in 5% non-fat milk and then incubated with primary antibodies, including anti-CTRP3 (1:1500, orb182796, Biorbty Ltd), anti-LAMP1 (1:1000, DF7033, Affinity), anti-JIP2 (1:750, DF3260, Affinity), anti-p-JNK (1:1000, AF3318, Affinity), anti-JNK (1:1000, AF6318, Affinity) and anti-GAPDH (1:20000, 10494-AP, Proteintech) at 4°C overnight. The membranes were incubated with horseradish peroxidase (HRP)-conjugated goat anti-rabbit antibody (1:10000, 19003-1-AP, Proteintech). GAPDH was used as an internal control. The signals were detected by using an ECL kit (Pierce Biotech, Rockford, IL, USA). Subsequently, the protein bands were analyzed with Image J 1.43 software.

### Measurement of ROS and MDA production

The production of ROS was detected by using Dichlorofluorescein diacetate (DCFH-DA) assay. Briefly, cells were incubated with 10 μmol/L DCFH-DA at 37°C in the dark for 20 min. Then, the cells were washed twice with PBS, and the fluorescence intensity was detected by using a fluorescence spectrophotometer (BioTek).

H9C2 cells were seeded in 96-well plates at a density of 1 × 10^4^ cells/well. Myocardial tissues were homogenized and the supernatant was obtained. The activity of MDA was measured by using MDA activity kit (Nanjing Jiancheng Bioengineering Institute, Nanjing, China).

### Analysis of cardiac troponin (cTn-I) release

H9C2 cell culture medium and myocardial tissue abrasive fluid were collected by centrifugation at 4°C for 15 min. The concentration of cTn-I in the supernatant medium was determined by using enzyme-linked immunosorbent assay kits (ELISA, Jiancheng, Nanjing, China) according to the manufacturer’s instructions. The absorbance of the samples was measured at 450 nm with a standard microplate reader (BioTek Instruments, Inc.).

### Co-immunoprecipitation (Co-IP) assay

Cells were lysed by utilizing Co-IP lysis buffer containing a protease inhibitor (Invitrogen, Carlsbad, USA). The lysates were collected and mixed with 2 μg of primary antibody or immunoglobulin G (IgG). Then, the mixture was incubated with protein A/G beads overnight at 4°C. The beads were washed three times and boiled in loading buffer for 5 min. All samples were analyzed by Western blotting using various antibodies.

### Animals and *in vivo* myocardial I/R injury model

C57BL/6J mice (weight 17–25 g) were purchased from Animal Laboratory of the Fourth Military Medical University and all mice were kept in a temperature and humidity-controlled room (25 ± 2°C, 60%–80%). Mice were randomly divided into 3 groups (*n* = 8 mice of each group): Sham group, I/R+ lentiviral vectors (LVs) group and I/R+ CTRP3 overexpressed lentiviral vectors (LV-CTRP3) group. Three days before operation, mice myocardium was injected with LV-CTRP3 (2 mg/kg). Mice were anesthetized by inhalation of 2% isoflurane, and an oblique incision was made in their left chest wall skin to reveal the heart. The left anterior descending coronary artery was ligated with a polypropylene 6–0 suture. After 30 min occlusion, the sutures were loosened for 24 h to establish the myocardial I/R model. Sham operated mice underwent left chest wall incision surgery, but the left anterior descending coronary artery was not ligated. This study was approved by the ethics committee of the Affiliated Hospital of Yanan University. All animal experimental procedures were in accordance with the guide for the care and use of laboratory animals of the National Institutes of health.

### Echocardiographic assessment

At the end of reperfusion, mice were immobilized on the experimental table, and ventricular function was examined by using an echocardiography system (Sequoia ACUSON, Siemens, Erlangen, Germany). Left ventricular end systolic diameter (LVESD), left ventricular end diastolic diameter (LVEDd), left ventricular end systolic volume (LVSV), and left ventricular end diastolic volume (LVDV) were measured and averaged over 3 consecutive cardiac cycles. As indicators of cardiac function, the left ventricular ejection fraction (LVEF) and left ventricular fractional shortening (LVFS) values were converted using the Simpson method with the following formulas: LVEF = (LVDV-LVSV)/LVDV × 100%; LVFS = (LVDD-LVSD)/LVDD × 100%. Experiments were performed 3 times and averaged.

### Infarct size determination

Mice were anesthetized with inhalation of 2% isoflurane after 24 h of reperfusion, and the hearts were subsequently excised and frozen for 30 min. Heart tissues were cut into 1-mm-thick sections and incubated with 1% 5-triphenyltetrazolium chloride (TTC) at 37°C for 15 min, with white being the infarct zone (INF) and red and white areas being the high-risk zone (AAR). Infarct size was expressed by using the following formula: Infarct size = INF/AAR × 100%.

### TUNEL staining

TUNEL assay was used to detect apoptosis in heart tissues. Myocardial sections were prepared and then were incubated with 50 μL TUNEL mixture at 37°C for 1 h. After washing with PBS, sections were stained with DAPI. Myocardial apoptosis was observed by fluorescence microscopy. The apoptotic rate was calculated as the number of apoptotic cells (green)/the number of total cells (blue) × 100%.

### Statistical analysis

Data were presented as the mean ± SEM. SPSS 23.0 software (SPSS, Chicago, IL, USA) was used to perform all analyses. Student’s *t*-test was performed for comparison between two groups. ANOVA was used to compare with multiple groups. A *P*-value of <0.05 was considered statistically significant.

### Ethics statement

Animal studies were performed in compliance with the ARRIVE guidelines. All animals received humane care according to the National Institutes of Health (USA) guidelines.

## RESULTS

### The expression of CTRP3 was downregulated in cardiomyocytes in response to OGD/R conditions

**Figure 1:**
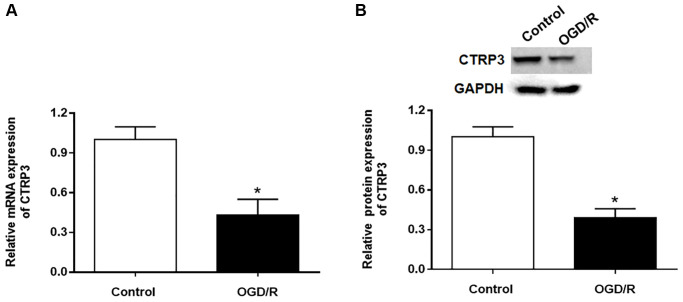
**Effect of OGD/R treatment on CTRP3 expression in H9C2 cells.** (**A**, **B**) Relative mRNA and protein expression of CTRP3 was determined by using RT-qPCR and Western blotting. ^*^p < 0.05 and the same as below.

To explore the role of CTRP3 in myocardia I/R injury, we assessed the expression of CTRP3 in H9C2 cells after OGD/R treatment by using RT-qPCR and Western blotting. The results showed that the mRNA expression of CTRP3 was significantly decreased in H9C2 cells in response to OGD/R treatment (Figure 1A, *P* < 0.05). Similarly, the protein level of CTRP3 was also remarkably restrained in H9C2 cells in response to OGD/R treatment (Figure 1B, *P* < 0.05).

### CTRP3 protected against OGD/R injury in H9C2 cells

**Figure 2:**
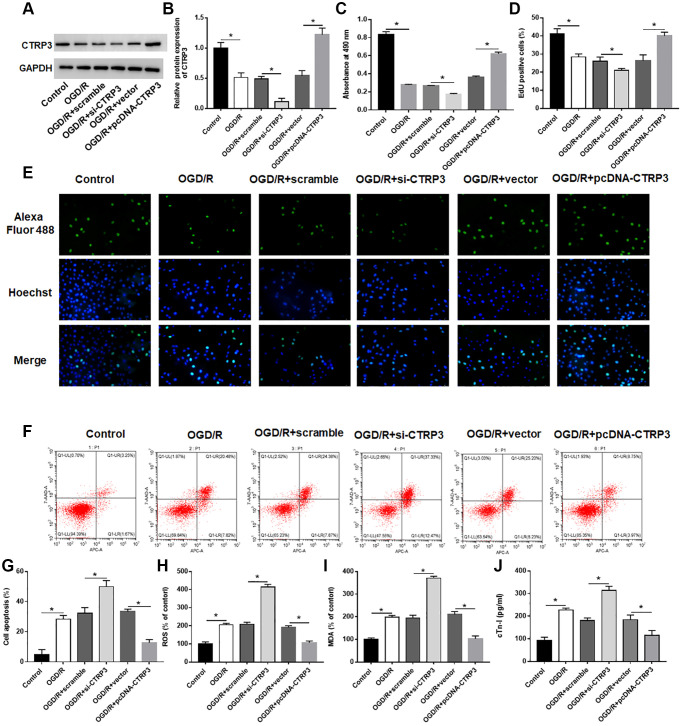
**Effect of CTRP3 on OGD/R injury in H9C2 cells.** H9C2 cells were transfected with pcDAN3.1-CTRP3 and CTRP3 siRNA or negative control (scramble and vector) to incubate for 24 h and then exposed to OGD/R injury. (**A**, **B**) Relative protein expression of CTRP3 was detected with Western blotting. (**C**–**E**) Effect of CTRP3 overexpression and inhibition on cell proliferation was assessed with CCK-8 and EdU assays. (**F**, **G**) Effect of CTRP3 overexpression and inhibition on cell apoptosis was detected with flow cytometry. (**H**–**J**) Effect of CTRP3 overexpression and inhibition on ROS, MDA and cTn-I production were determined.

To investigate the biological effect of CTRP3 in OGD/R injury, H9C2 cells were transfected with pcDNA-CTRP3 or si-CTRP3. The results of Western blotting indicated that transfection of pcDNA-CTRP3 was efficient to elevate CTRP3 protein level, and the introduction of CTRP3 siRNA caused a markedly decrease of CTRP3 level in H92C cells (Figure 2A and 2B, *P* < 0.05). MTT and EdU assay results showed that CTRP3 overexpression accelerated the growth of H9C2 cells after OGD/R treatment, while silence of CTRP3 inhibited cell proliferation (Figure 2C–2E, *P* < 0.05). In addition, flow cytometry verified that the apoptosis ability of H9C2 cells was notably suppressed after CTRP3 overexpression, while increased after CTRP3 silence (Figure 2F and 2G, *P* < 0.05). Moreover, upregulation of CTRP3 reduced ROS, MDA and cTn-I content, and silencing CTRP3 notably facilitated ROS, MDA and cTn-I production in H92C cells after OGD/R treatment (Figure 2H–2J, *P* < 0.05).

### LAMP1 was a receptor of CTRP3

**Figure 3:**
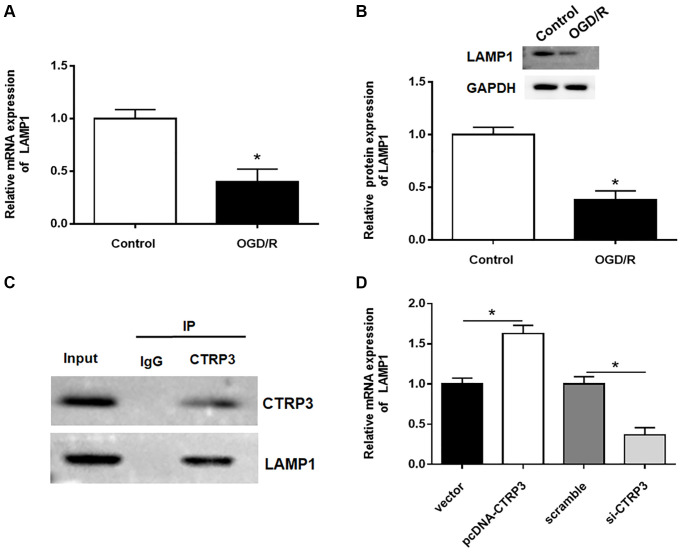
**CTRP3 directly interacted with LAMP1.** (**A**, **B**) Relative mRNA and protein expression of CTRP3 was determined by using RT-qPCR and Western blotting. (**C**) The relationship between CTRP3 and LAMP1 was detected by using Co-IP. (**D**) The effects of CTRP3 silence or overexpression on the mRNA level of LAMP1 was measured by using RT-qPCR.

To further detect how CTRP3 participated in OGD/R injury, proteins that could interact with CTRP3 were investigated. LAMP1 has been reported to be a putative receptor of CTRP3 [22], hence, the expression of LAMP1 in H9C2 cells after OGD/R treatment was assessed. The results of RT-qPCR and Western blotting showed that the mRNA and protein levels of LAMP1 were remarkably downregulated in H9C2 cells (Figure 3A and 3B, *P* < 0.05). Subsequently, the interaction between CTRP3 and LAMP1 was validated by using Co-IP assay (Figure 3C). Besides, the upregulation of CTRP3 caused a significant increase of LAMP1 protein level in H9C2 cells, whereas knockdown of CTRP3 led to a opposite result (Figure 3D, *P* < 0.05).

### Upregulation of LAMP1 attenuated OGD/R injury in H9C2 cells

**Figure 4:**
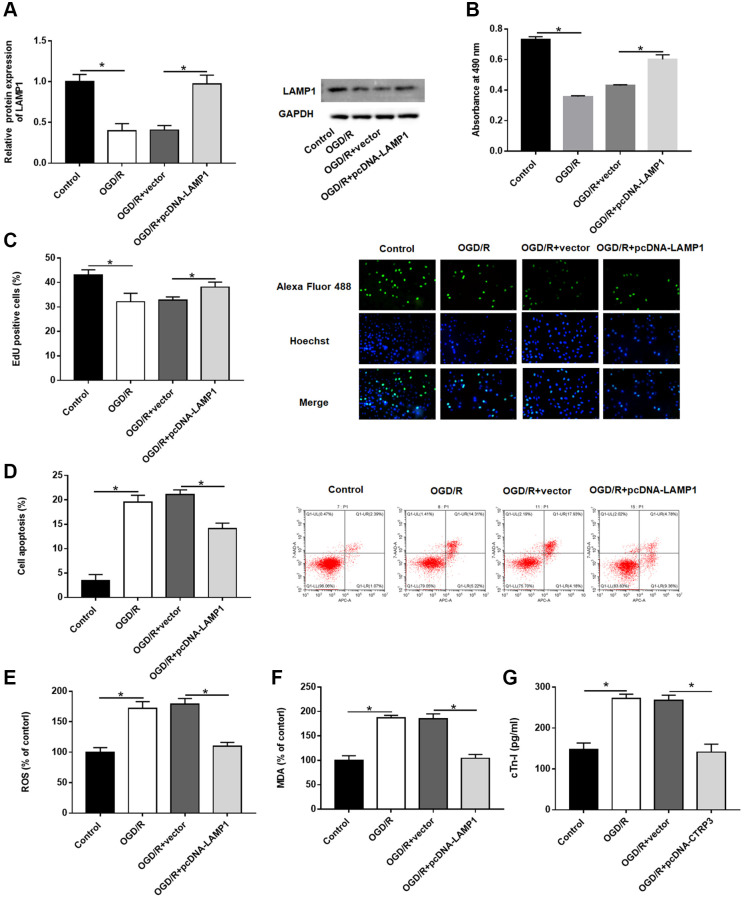
**Effect of LAMP1 overexpression on OGD/R injury in H9C2 cells.** H9C2 cells were transfected with pcDNA-LAMP1 or negative control (vector) for 24 h and then exposed to OGD/R injury. (**A**) Relative protein expression of LAMP1was detected by Western blotting. (**B**, **C**) Effect of LAMP1 overexpression on cell proliferation was assessed by using CCK-8 and EdU assays. (**D**) Effect of LAMP1 overexpression on cell apoptosis was detected by flow cytometry. (**E**–**G**) Effects of CTRP3 overexpression on ROS, MDA and cTn-I production were determined.

Then, the effect of LAMP1 in H9C2 cells was investigated. The protein level of LAMP1 was notably increased after H9C2 cells transfected with pcDNA-LAMP1 compared with OGD/R treatment (Figure 4A, *P* < 0.05). LAMP1 overexpression significantly promoted cell proliferation after OGD/R injury by using MTT and EdU assays (Figure 4B and 4C, *P* < 0.05). Moreover, cell apoptosis following OGD/R injury was conspicuously reduced by LAMP1 overexpression (Figure 4D, *P* < 0.05). Likewise, upregulation of LAMP1 markedly cut down the production of ROS, MDA and cTn-I in H9C2 cells after OGD/R treatment (Figure 4E–4G, *P* < 0.05). These data indicated that LAMP1 overexpression improved OGD/G injury in H9C2 cells.

### LAMP1 overexpression abolished the biological functions of CTRP3 silence in H9C2 cells

**Figure 5:**
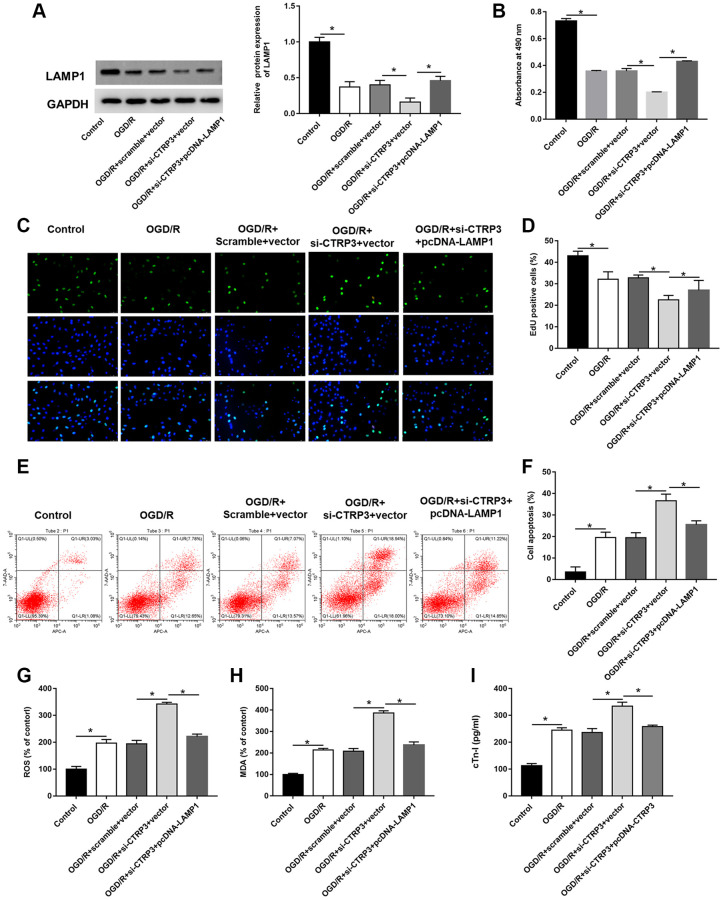
**Overexpression of LAMP1 reversed the inhibitory effect of CTRP3 silencing on OGD/R injury in H9C2 cells.** H9C2 cells were transfected with CTRP3 siRNA alone or together with pcDNA-LAMP1 for 24 h and then exposed to OGD/R injury. (**A**) The protein expression of LAMP1 was examined with Western blotting. (**B**–**D**) Cell proliferation was detected by using MTT and EdU assays. (**E**, **F**) Cell apoptosis ratio was measured by using flow cytometry. (**G**–**I**) The production of ROS, MDA and cTn-I was determined.

To verify whether CTRP3 regulated the biological functions of H9C2 cells dependent on LAMP1 during OGD/R injury, H9C2 cells were transfected with CTRP3 siRNA alone or together with pcDNA-LAMP1. Silencing CTRP3 significantly reduced the protein expression of LAMP1, attenuated the proliferation ability of H9C2 cells, and LAMP1 overexpression reversed this alteration caused by CTRP3 knockdown (Figure 5A–5D, *P* < 0.05). Flow cytometry analysis demonstrated that enforced expression of LAMP1 could restore the CTRP3 deficiency-mediated cell apoptosis (Figure 5E, 5F, *P* < 0.05). Furthermore, absence of CTRP3 promoted ROS, MDA and cTn-I production in H92C cells subjected to OGD/R, which was markedly countervailed by LAMP1 overexpression (Figure 5G–5I, *P* < 0.05).

### CTRP3 conferred its protective effects on H9C2 cells via LAMP1/JIP2/JNK pathway

**Figure 6:**
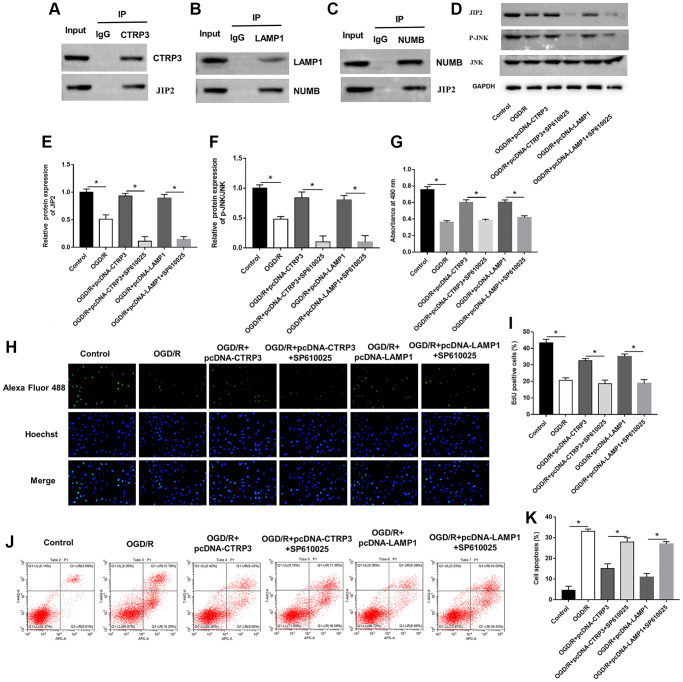
**CTRP3 and LAMP1 overexpression accelerated activation of JIP2/JNK signaling pathway.** (**A**) The relationship between CTRP3 and JIP2 was detected with Co-IP. (**B**, **C**) The relationships among LAMP1, NUMB and JIP2 were detected by using Co-IP. (**D**–**F**) Relative protein expression of JIP2 and p-JNK was detected with Western blotting. (**G**–**I**) Cell proliferation was detected by using MTT and EdU assays. (**J**, **K**) Cell apoptosis ratio was measured by using flow cytometry.

The JNK pathway is known to associate with myocardial I/R injury and JNK-interacting protein-2 (JIP2) is closely related to JNK. BioGRID website (https://thebiogrid.org) and MGnify website (http://www.ebi.ac.uk/metagenomics) predicted that CTRP3 may serve as a binding partner of JIP2. Furthermore, Co-IP assay suggested that CTRP3 physically interacted with JIP2 (Figure 6A). More interestingly, the HitPredict website (http://www.hitpredict.org/) found that LAMP1 could bind to protein numb homolog (NUMB), which in turn interacted with JIP2. Co-IP confirmed the association between LAMP1, NUBM and JIP2 (Figure 6B and 6C). Subsequently, we explored the possible role of the JIP2/JNK pathway in CTRP3-related molecular events in H9C2 cells during OGD/R injury. Western blot assay results showed that the protein levels of JIP2 and p-JNK were significantly reduced after OGD/R treatment, while increased after H9C2 cells transfected with pcDNA-CTRP3 and pcDNA-LAMP1 (Figure 6D–6F, *P* < 0.05). Moreover, the effects of SP600125 on H9C2 cell proliferation and apoptosis were also investigated. JNK inhibitor (SP610025) reversed the promotive effects of CTRP3 or LAMP1 overexpression on the upregulation of JIP2 and p-JNK expression (Figure 6D–6F, *P* < 0.05). Overexpression of CTRP3 and LAMP1 effectively increased cell proliferation and decreased cell apoptosis, while SP610025 treatment could reverse these effects (Figure 6G–6K, *P* < 0.05). These results proved that CTRP3 alleviated OGD/R injury via LAMP1/JIP2/JNK pathway.

### Overexpression of CTRP3 ameliorated I/R injury

**Figure 7:**
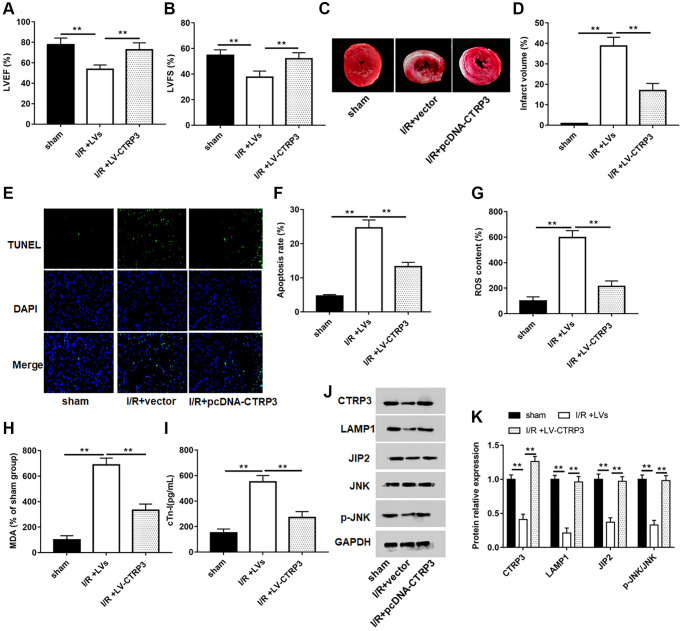
**Overexpression of CTRP3 ameliorated I/R injury.** (**A**, **B**) The levels of LVEF and LVFS were detected by echocardiograph as indexes for cardiac function at the end of the 24 h reperfusion. (**C**) Representative images of stained myocardial tissues of mice in all groups. (**D**) The percentage of myocardial infarction area of all groups. (**E**, **F**) Cardiomyocyte apoptosis was determined by using TUNEL assay. (**G**–**I**) The production of ROS, MDA and cTn-I was determined. (**J,** **K**) Western blotting was performed to analyze the protein expression of CTRP3, LAMPA, JIP2, JNK and p-JNK in myocardial tissue.

A mouse model of myocardial I/R was established to explore the role of CTRP3 on myocardial injury. The levels of LVEF and LVFS (Figure 7A and 7B, *P* < 0.05), and protein expression of CTRP3, LAMP1, JIP2 and p-JNK (Figure 7J and 7K, *P* < 0.05) were significantly lower in mice subjected to myocardial I/R surgery. Moreover, myocardial infarct volume (Figure 7C and 7D, *P* < 0.05), apoptosis (Figure 7E and 7F, *P* < 0.05), and the contents of ROS (Figure 7G, *P* < 0.05), MDA (Figure 7H, *P* < 0.05) and c-Tn1 (Figure 7I, *P* < 0.05) were increased after I/R injury. However, mice injected to LV-CTRP3 could reverse the inhibitory effects of I/R injury on LVEF and LVFS levels and protein levels of CTRP3, LAMP1, JIP2 and p-JNK, and the promotive effects on myocardial infarct volume, apoptosis, and the contents of ROS, MDA and c-Tn1 (*P* < 0.05).

## DISCUSSION

Myocardial infarction is a severe disease associated with high mortality, and reestablishing the blood supply to the ischemic myocardial tissue is the most feasible treatment. However, prone to myocardial I/R injury is prone to occur [23]. Previous studies have shown that myocardial infarction prevention and treatment could target myocardial protective genes or deleterious genes or interfere with pathway gene expression, prompting the balance of protective genes and deleterious gene expression and regulating up- and downstream pathways [24]. CTRP3 was reported to be associated with the development of several heart related disorders. Overexpression of CTRP3 could enhance the therapeutic efficacy of mesenchymal stromal cells in myocardial infarction [25]. However, CTRP3 was upregulated in cardiac hypertrophy, and overexpression of CTRP3 aggravated cardiac hypertrophy and cardiac dysfunction [26]. Therefore, we speculated that the different roles of CTRP3 might be associated with the pathological process of different diseases. CTRP3 inhibited high glucose induced oxidative stress, inflammation and apoptosis, and attenuated cardiomyocyte death and improved cardiac function in streptozotocin treated rats [15]. Zhang et al. suggested that CTRP3 was an endogenous regulator of mitochondrial biogenesis, which could protect cardiomyocytes by improving mitochondrial dysfunction [27]. In present study, CTRP3 was downregulated in OGD/R treated H9C2 cells and myocardial I/R injury mice. Overexpression of CTRP3 predominantly promoted H9C2 cell proliferation and restrained apoptosis and oxidative stress, whereas CTRP3 knockdown led to opposite results, illustrating that CTRP3 played a positive role in myocardial I/R. Moreover, overexpression of CTRP3 improved left ventricular function, attenuated myocardial infarct size and apoptosis, and suppressed ROS, MDA and cTn-I levels in mice subjected to I/R injury compared with mice in the sham group, indicating that CTRP3 has a protective effect on mice subjected to myocardial I/R injury.

Current evidence confirmed that LAMP1 was a routinely used as a lysosome marker and closely correlated with autophagy [28]. Upregulation of LAMP1 could form autolysosomes and accelerate ROS removal, thus inhibiting oxidative stress [29]. It is well known that excessive superoxide mediated oxidative stress and autophagy plays an important role in myocardial injury [30], suggesting that LAMP1 may be a key player in cardiomyocytes. Hence, whether CTRP3 and LAMP1 are related needs to be discussion. Studies found that CTRP3 directly inhibited the inflammatory response of psoriatic keratinocytes *in vitro* by blocking the phosphorylation of signal transducer and activator of transcription 3 by LAMP1 [31]. Our study revealed that CTRP3 attenuated apoptosis and oxidative stress in H9C2 cells after OGD/R stimulation via upregulating LAMP1. Next, Co-IP assay was performed to validate the relationship between CTRP3 and LAMP1. Results as we speculated, CTRP3 ameliorated myocardial injury caused by OGD/R treatment via LAMP1. Overexpression of LAMP1 remarkably reversed the inhibitory effects of CTRP3 silence on H9C2 cell proliferation, and the promotive effects on cell apoptosis and oxidative stress.

NUMB plays an important role in cardiac progenitor cell differentiation and cardiac morphogenesis and serves as a potential candidate gene for cardiac regeneration and treatment of congenital heart disease [32, 33]. Mitogen-activated protein kinase (MAPK) signaling pathway is associated with multiple cardiac diseases, including cardiac fibrosis, myocardial I/R injury [34]. JIP2 is a key scaffolding protein that promotes the activity of MAPK pathway proteins, including JNKs, and plays an important role in myocardial I/R injury [35]. NUNB acts as an intermediate connector protein of LAMP1 regulating the JIP2/JNK pathway. In addition, CTRP3 also protects against myocardial I/R injury by interacting with JIP2.

JNK pathway was involved in cellular fate such as proliferation, migration, invasion and apoptosis [36, 37]. However, the role of JNK pathway in myocardial I/R injury is controversial. Tumor necrosis factor receptor-associated factor 1 (TRAF1) aggravated the development of myocardial I/R injury by enhancing the activation of the JNK pathway [38]. However, alamandine pretreatment alleviates pathological alterations and cell injury in myocardium by activating JNK phosphorylation [39]. Cao et al. showed that activation of JNK increased the expression of heme oxygenase-1 (HO-1), which could reduce ROS-induced cell death in myocardial I/R injury [40]. Our results suggested that overexpression of CTRP3 or LAMP1 increased JIP2 and p-JNK protein levels in H9C2 cells after OGD/R treatment, thereby activating JIP2/JNK pathway to promote H9C2 cell proliferation and suppress cell apoptosis, which could be abolish by SP600125.

## CONCLUSION

In conclusion, the present study highlighted the protective roles of CTRP3 in myocardial I/R injury, manifested by promoting cell proliferation and suppressing cell apoptosis and oxidative stress via upregulating LAMP1 and activating JIP2/JNK signaling pathway. Therefore, CTRP3 may be a promising therapeutic target for the treatment of myocardial I/R injury.
